# 

**Published:** 1891-11

**Authors:** 


### CONTENTS:

Article I.—On Some Minor Surgical Operations Performed in or about the Mouth. By F. Breese. - - - - 289
" II.—La Grippe—Origin, History, Treatment. - - 303
" III.—Diseases of the Human Body which have been traced to the Action of Mouth-Bacteria. By W. D. Miller, M. D., Ph. D. - - - - - - 311
" IV.—The Care of Children's Teeth. - - - - 319
" V.—Finishing the Margins of Teeth. - - - - 324

### COMMUNICATIONS.

American Academy of Dental Science. - - - - - - 327
Maryland State Dental Association. - - - - - - 328

### MONTHLY SUMMARY.

Is Dentistry a Dangerous Profession. - - - - - 328
Dentistry in France and America. - - - - - - 330
Making an Artificial Set of Teeth. - - - - - - 332
Saving a Nerve. - - - - - - - - - 334
Was Cocaine the Cause of this Death. - - - - - 335
Happy Thought. - - - - - - - - - 336

